# Workplace Impact of Menopause Symptoms Among Canadian Women Physicians

**DOI:** 10.3390/healthcare13212699

**Published:** 2025-10-25

**Authors:** Shannon E. Brent, Lindsay Shirreff, Natalie L. Yanchar, Marie Christakis

**Affiliations:** 1 Temerty Faculty of Medicine, University of Toronto, 2109 Medical Sciences Building, 1 King’s College Cir, Toronto, ON M5S 3K3, Canada; 2 Department of Obstetrics and Gynaecology, University of Toronto, 123 Edward Street, Suite 1200, Toronto, ON M5G 1E2, Canadamarie.christakis@sinaihealth.ca (M.C.); 3 Department of Obstetrics and Gynaecology, Mount Sinai Hospital, 700 University Ave, Toronto, ON M5G 1Z5, Canada; 4 Department of Surgery, University of Calgary, Foothills Medical Centre, 1403 29 St NW, Calgary, AB T2N 2T9, Canada

**Keywords:** menopause, physician wellbeing, mature women’s health, occupational health

## Abstract

Background/Objectives: Menopause is a significant, universal hormonal transition, with symptoms impacting ~80% of women. Research shows that menopause can be professionally disruptive, contributing to decreased productivity, absenteeism, and early exit from the workplace. The objective of this study was to describe the landscape of menopause among Canadian women physicians and explore its potential impact on work performance, job satisfaction, and absenteeism. Methods: In this exploratory cross-sectional study, Canadian physicians self-identifying as women and peri-menopausal or menopausal were invited to participate in an online survey between May–September 2023. Demographic and practice characteristics data were collected. A modified Menopause Rating Scale (MRS) was used to quantify symptom burden. Qualitative data describing the menopausal experience were collected as well. Primary outcome was self-reported work performance. Secondary outcomes included perceived impact of menopause on promotional opportunities, absenteeism, and job satisfaction. Multivariable regression was used to examine associations between MRS scores and outcomes of interest. Results: Among 217 respondents, 47.7% reported a severe menopausal symptom burden; 40% felt menopause negatively impacted work performance, and 16.1% expressed job dissatisfaction. However, fewer than 10 respondents (4.6%) ever took time off for menopausal symptoms. Increasing MRS scores were significantly associated with negative perceived work performance (*p* < 0.001), fewer promotional opportunities (*p* < 0.001), and lower job satisfaction (*p* = 0.006) when controlling for confounders. Qualitative responses were provided by 43 participants, 6 of whom reported positive aspects of the menopausal transition, whereas 20 elaborated on the challenges. Conclusions: Canadian women physicians can experience severe menopausal symptoms, often without support. This needs assessment highlights an important occupational health issue and suggests that opportunities remain for medical institutions and employers to formally recognize and study this life stage of women physicians to improve well-being for this valuable workforce.

## 1. Introduction

Menopause is a universal and significant hormonal transition marked by the cessation of menses, typically between the ages of 45 and 55 [1]. Nearly 80% of women have menopausal symptoms, which can include vasomotor symptoms (night sweats and hot flashes), poor concentration, sleep disturbances, poor memory, and mood changes. Symptoms associated with the menopausal transition can be disruptive, both professionally and personally, and can lead to decreased quality of life [2].

The menopausal transition lasts an average of 7 years [3] and, given its typical timing, can often coincide with the most productive and formative years in a woman’s career [4,5]. The impact of the menopausal transition on work performance in several occupations has been explored. Studies among nurses, teachers, and corporate workers have demonstrated that many women who work throughout the menopausal transition perceive negative effects on productivity [6], driven by not only the symptoms of menopause, but also by the physical characteristics and psychosocial culture of the workplace [7,8,9]. Increased absences [10] and early exit from the workplace [11], owing to menopausal symptoms, have also been demonstrated. A study of academic physicians in Egypt reported a high burden of menopausal symptoms and lack of institutional support [9]. Existing evidence has prompted calls to acknowledge menopause as an occupational health issue and promote an inclusive culture [12,13].

The impact of the menopausal transition on women physicians in Canada, and the subsequent impact on the healthcare workforce and delivery, has not been examined. A recent CMAJ editorial by Shirreff and Christakis emphasized the importance of examining this in the context of the Canadian healthcare system, where women physicians make up nearly half of the current workforce across Canada, and nearly one quarter of women physicians are between the ages of 45–54 [14]. As such, understanding the potential burden of the menopausal transition in the workplace and identifying supports warrants exploration. The objective of this needs assessment study was to explore the lived experience and impact of the menopausal transition in the workplace among women physicians in Canada. We sought to describe the landscape of menopause in the Canadian medical community, quantify its potential impact on work performance and absenteeism, in a way that might inform future workplace initiatives aimed at promoting age- and gender-equity to maximize supports.

## 2. Materials and Methods

### 2.1. Study Design, Setting, and Participants

In this cross-sectional, exploratory needs-assessment study, an online survey tool was developed to collect observational data on the impact of the menopausal transition in the workplace among women physicians in Canada. The content of the survey tool was informed by existing survey-based research on menopause in the workplace [7,9,15]. Menopausal symptoms were quantified using a modified Menopause Rating Scale (MRS) [16], a standardized and formally validated health-related quality of life tool. The MRS was modified to capture symptoms retrospectively, prompting participants to report symptom severity at its worst during the menopausal transition. The MRS tool can be accessed at http://www.menopause-rating-scale.info/ (accessed on 4 November 2022) [16].

The population of interest was physicians working in Canada who self-identified as female. Menopausal status was determined by self-report. Those identifying as perimenopausal or postmenopausal were included; pre-menopausal participants were excluded. Other exclusion criteria included male sex, those not currently employed as a physician, and those not working in Canada. Participants who voluntarily exited the survey after these initial screening questions were also excluded.

The online survey was distributed online from May to September 2023. The study team contacted several women-focused professional societies across Canada for study distribution. However, these organizations stated survey distribution was outside the scope of their mandate, and thus, we were unable to access this population at a national level. As such, in an attempt to access a representative sample of female physicians, the survey tool was disseminated broadly and electronically via Canadian membership-based third-party organizations (e.g., academic medical departments/faculties, provincial membership-based organizations) spanning several medical specialties, and via social media. Participants were also invited to share the survey link with colleagues to maximize the number of respondents.

Participation in this study was voluntary and confidential. Participants were informed that their response to the survey implied consent to participate, and all responses were anonymized. Approval was obtained from the University of Toronto Health Sciences Research Ethics Board.

### 2.2. Outcomes and Covariates

Quantitative data were collected on participant demographics, province of employment, menopausal status (self-reported/self-identified), menopausal symptoms, characteristics/conditions of workplaces, specialty (surgical vs. non-surgical), self-reported work performance, absenteeism, perceptions of menopause, use of hormone therapy, and presence/absence of workplace supports. Qualitative data were also collected regarding the experience of menopausal symptoms and workplace adjustments that participants perceived might be helpful during the menopausal transition. At the end of the survey, participants were invited to share other open-ended comments about their menopausal transition. Selected representative open-ended responses were compiled as quotes.

The main quantitative exposure of interest was burden of menopausal symptoms when participants recalled symptoms being at their worst, as measured by the MRS. Participants provided self-reported severity scores for each of 11 symptoms of menopause, rated from 0 (none) to 4 (very severe). The cumulative MRS score was used as the exposure variable, with higher total scores corresponding to a higher burden of menopausal symptoms. A cumulative MRS score of ≥16 was considered “severe” [17]. The primary outcome was self-reported work performance as measured by a 5-point Likert scale, with 1 being “strongly disagree” that performance at work has been/was negatively affected by menopausal symptoms, and 5 being “strongly agree”. Secondary outcomes included perceived impact on promotion/leadership opportunities, where higher scores on a 5-point Likert scale corresponded to menopausal symptoms having a perceived greater negative impact on promotion, absences from work (binary outcome), and job satisfaction. Higher scores on a 7-point Likert scale corresponded to higher work satisfaction. All outcomes were based on self-report.

Covariates of interest included age, use of menopausal hormone therapy (MHT), overnight call, specialty, type of practice, time since menopause, presence of formal institutional support, job satisfaction, and flexible working hours.

### 2.3. Statistical Analysis

Descriptive statistics were computed to summarize all study variables. Covariates of interest were identified a priori. Multivariable linear regression analyses were used to examine the relationship between cumulative MRS score and the outcomes of interest: work performance, promotional opportunities, absences, and job satisfaction. Models were built to assess the influence of possible confounders/covariates. Beta coefficients were computed to quantify the association between the cumulative MRS score and the outcome of interest. A level of significance of 0.05 was used for all inferential analyses, with *p*-values < 0.05 reported as statistically significant. All statistical analysis was conducted using SPSS Statistics version 29.

### 2.4. Qualitative Analysis

Qualitative responses were screened and aggregated where appropriate to identify common themes.

## 3. Results

**Figure 1:**
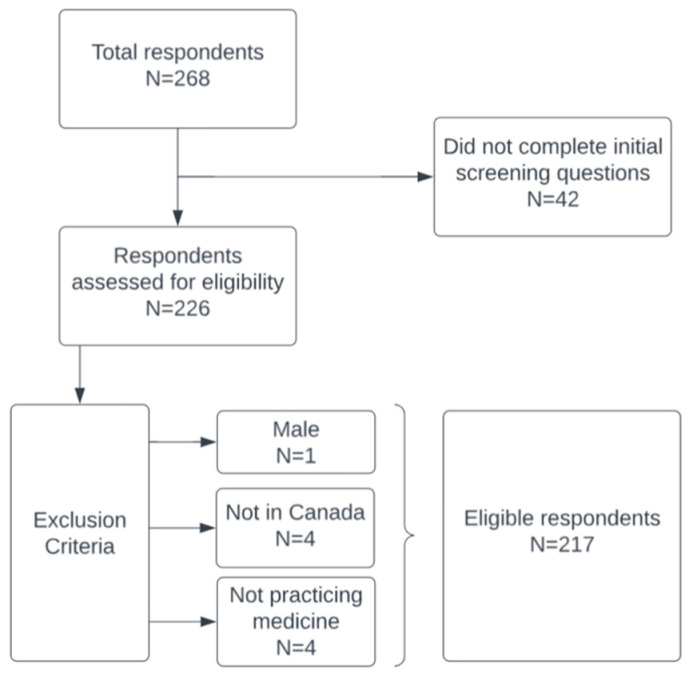
Flow chart of inclusion and exclusion criteria and number of study participants.

Between 8 May and 21 September 2023, 268 individuals initiated the survey. After exclusions, 217 responses remained for analysis (Figure 1).

### 3.1. Participant Characteristics

Participant characteristics, including demographics, geographic location, menopausal status, and age of last menstrual period, are summarized in Table 1. Most respondents were between the ages of 46–54 (N = 101, 46.5%) or 55–64 (N = 77, 35.5%). While all regions in Canada were represented, most respondents lived in either Western Canada (N = 93, 42.9%) or Ontario (N = 89, 41.0%). Most participants were postmenopausal (N = 156, 71.9%), with the remainder being perimenopausal (N = 61, 28.1%).

### 3.2. Workplace and Practice Characteristics

Practice characteristics are summarized in Table 2. Most respondents worked in non-surgical specialties (N = 149, 68.7%), worked overnight call/shifts (N = 127, 58.5%) and worked as part of a practice group (N = 155, 71.4%). Most reported having flexible working hours (N = 136, 62.7%). On a 7-point Likert scale, a minority of respondents (16.1% (N = 35)) were dissatisfied, very dissatisfied, or extremely dissatisfied with their job, and an additional 13.4% (N = 29) were neither satisfied nor dissatisfied. The overall mean job satisfaction was 4.86 (SD +/− 1.187) on a scale of 1–7.

### 3.3. Menopausal Symptoms and MHT Use

**Figure 2:**
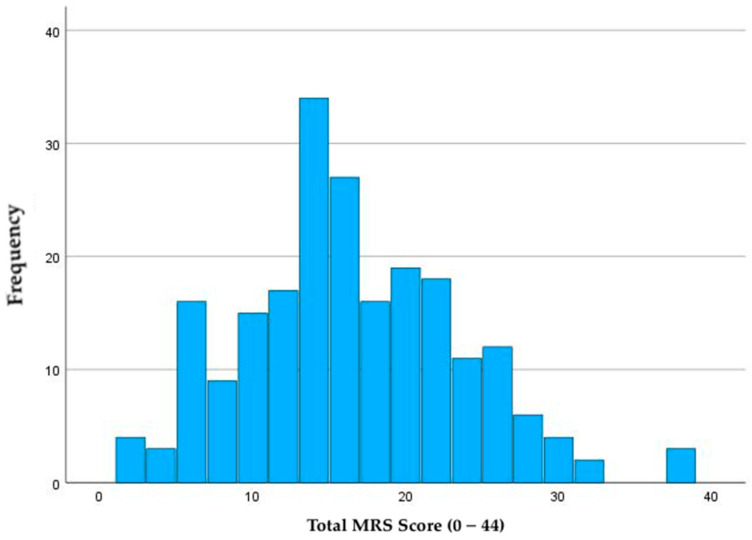
Distribution of total cumulative Menopause Rating Scale score among Canadian women physicians.

The distribution of the burden of menopausal symptoms among our study population is shown in Figure 2. Mean cumulative MRS score was 15.9 (SD 7). Over 47% of participants had cumulative MRS scores that were considered “severe” (N = 103, 47.7%). Of the 186 respondents who endorsed hot flashes (HF) at work (N = 186), approximately 24% experienced 6 or more HF per day at work, and 45.6% of respondents stated HF were problematic at work during the worst point of their menopausal transition. The most frequent symptoms felt to be problematic for work were related to sleep (N = 112, 51.6%), physical and mental exhaustion (N = 116, 53.5%), and irritability (N = 106, 48.8%). The most common work situations that made coping with HF more difficult included hot/unventilated workspaces (N = 113, 52.1%), high visibility work (e.g., presentations, teaching; N = 58, 26.7%), and hot/unventilated operating rooms (N = 46, 21.2%). Although not an offered response option, many respondents suggested personal protective equipment as an open-ended response when asked about factors that exacerbated HF at work (N = 7, 3.2%).

The prevalence of MHT use in our sample was 36.9% (N = 80). Of those participants who used MHT, 57.5% (N = 46) endorsed that the impact of their symptoms on performance at work contributed to the decision to use MHT, and 65% (N = 52) felt MHT helped improve perceived work performance.

### 3.4. Impact of Menopausal Transition on Work

Overall, approximately 40% (N = 88) of respondents felt that their work performance was negatively affected by menopausal symptoms (agree: 31.3%; strongly agree: 9.2%). A majority (N = 136, 62.6%) of respondents felt their menopausal symptoms could have affected work performance, but they worked very hard to overcome and maintain performance (agree: 35.9%; strongly agree 26.7%).

Approximately 13% (N = 28) felt their menopausal symptoms negatively impacted opportunities for promotion or leadership. Only 10 respondents (4.6%) reported ever taking time off because of their menopausal symptoms. Less than 1% of respondents (N = 2) received any formal institutional or employer support for their menopausal symptoms.

Simple linear regression showed that higher composite MRS scores were significantly associated with negative perceived work performance (ß = 0.086, 95% CI [0.068, 0.104], *p* < 0.001; Table 3). When controlling for possible confounders, including age, practice type, practice facility type, specialty type, overnight call, and flexible work hours, the relationship between increasing MRS score and negative perceived work performance remained statistically significant. Of these covariates, age 46–54 was the only significant predictor of perceived negative work performance.

### 3.5. Secondary Outcomes

Increasing composite MRS score was found to have a significant association with perceived negative impact on promotional opportunities (ß = 0.083, 95% CI [0.065, 0.101], *p* < 0.001; Table 4) when controlling for possible confounders. Flexible work was the only other statistically significant predictor of perceived negative impact on promotional opportunities.

Within univariate analysis, those with higher MRS scores were significantly more likely to take time off (OR 1.11, 95% CI [1.02, 1.121], *p =* 0.020). However, when controlling for covariates in multivariable analyses, this relationship was no longer statistically significant. Lastly, higher MRS scores were significantly associated with lower job satisfaction (ß = −0.024, [−0.047, −0.001], *p =* 0.039; Table 5) when controlling for possible confounders.

### 3.6. Qualitative Findings

Forty-three participants provided comments about the menopausal transition, representing a range of opinions; of these, 6 respondents highlighted positive aspects of the menopausal transition; 20 respondents elaborated on challenges associated with the menopausal transition. Key themes included challenges accessing medical care and fears of being perceived as weak by colleagues. A selection of quotes is presented in Box 1.

Box 1 **Open-ended responses on the experience of the menopausal transition, categorized by theme.**

**Access to Care**

*“I have sought out physicians and other resources for perimenopause but they are difficult to find even as a physician who knows the system”*
*“The worst was not being taken seriously by my own family doctor of more than 20 years”*
*“I suffered through the symptoms despite reaching out to my male GP about it. HRT was not offered until a few months ago when I saw a OBGYN for post-menopausal bleeding. After testing [was] normal, she offered HRT and I feel great. My sexual symptoms resolved, [I] sleep very well, and ‘arthritic’ pain disappeared.”*

**Workplace Supports**

*“I am sure directors and colleagues seldom know about personal medical or menopausal issues. I don’t share because I am sure it would be viewed as a weakness.”*
*“Generally something that women are expected to “tolerate”—Would not be understood or accommodated by men in my department.”*

**Severity of the Transition**

*“I had no idea how bad it can get. We need so much more education about this issue.”*
*“It’s worse than most people acknowledge”*
*“I had no idea that sleep disturbance would so profoundly affect me or be so difficult to treat.”*

**Positive Views of Menopausal Transition**

*“Tough it out is the way to go and just deal with it. There are far more challenging disabilities that others deal with.”*
*“It was a very easy experience and keeping working helped.”*
*“I think menopause was freeing experience for me. My symptoms were mild and I enjoy the lack of monthly cycle of pain and mood changes.”*

## 4. Discussion

Although a small sample, we observed a high burden of menopausal symptoms in our exploratory survey of women physicians. We also found an association between symptomatology and perceived negative work performance, and a perceived negative impact on promotional opportunities among respondents. Interestingly, multivariable regression analysis suggested that age 46–54 was the only significant independent predictor of perceived negative work performance. This may be a result of recency bias, as these participants were more likely to be experiencing the menopausal transition around the time survey response compared to other age groups, when discussion and awareness around menopause have become more common on a societal level as well.

We also observed a high rate of MHT use (36.9%) compared to estimates among the general Canadian population (~10%) [18]. Respondents found MHT helpful, although several respondents highlighted challenges in accessing medical care for menopausal concerns. We also observed a higher rate of job dissatisfaction among our sample (16.1%) compared to women physicians of all ages in Canada (14.8%) and male physicians of all ages in Canada (11.0%) [19]. Although our sample may not be representative of the entire woman physician workforce in Canada, our findings suggest there is nonetheless an ongoing need for further workplace supports for a subset of woman physicians during the menopausal transition.

Our findings align with previous research demonstrating that the menopause transition can pose specific challenges in the workplace [6,7,10,15]. However, there may be unique workplace and cultural factors that may further its impact on women physicians. Compared to previous reports on the menopausal transition among non-physician professional women [7,11,15], we observed a higher symptom burden among our women physician respondents, higher MHT use, and less time off. In several studies of professional women [7,11], flexible work hours and time off were reported as helpful and considered commonly used coping strategies. However, only 4.6% of our study participants ever took time off, and flexible work was the only statistically significant predictor of perceived negative impact on promotional opportunities. It is possible that healthcare workplace culture entails leadership positions subject to rigid demands. Further, women physicians seeking work schedule adjustments may be perceived as less committed and passed over for promotion. This, paired with stigma around speaking about menopause in the workplace [20], may be isolating physicians with bothersome symptoms.

In October 2023, the Menopause Foundation of Canada (MFC) released “Menopause and Work in Canada” [13], highlighting the economic impact of menopause and opportunities to support women to thrive during the menopausal transition. The report also highlighted unique challenges in nursing, including a need to “self-accommodate”, given a lack of workplace supports. Our findings reflect a similar lived experience among the women physicians in our sample, who work in parallel to nursing colleagues. In our study, most respondents described working harder to maintain performance, despite their symptoms. Furthermore, formal workplace/employer supports were virtually absent for our respondents. Without support, the impact of the menopausal transition on sleep, focus, and cognition could potentially pose an occupational health concern for physicians, given the importance of executive functioning in all types of practice. In recent years, within Canada, there has been a push to address various health concerns among physicians including burnout, mental health, and even added resources related to pregnancy. Perhaps the time has come to offer more formal support to women physicians experiencing a high burden of menopausal symptoms. Ideally, physicians ought to be given an opportunity to seek support and flexibility and have access to medical and/or occupational resources when facing health concerns.

### 4.1. Limitations

Our findings must be viewed in the context of the study design. Although our convenience sampling approach successfully captured responses across a diverse population of women physicians, our sample may over-represent physicians with a severe burden of menopausal symptoms, higher rates of MHT use, and better access to healthcare. Physicians who experienced more severe menopausal symptoms may have been more likely to self-select to participate in our study due to personal interest. Furthermore, this sample of 217 women physicians accounts for a small proportion of the approximately 12,000 women physicians in Canada undergoing menopause [14]. As such, these results may not be generalizable to the entire woman physician work-force, given the variety of experiences and work contexts; however, while this is likely not an adequate representation, our work does uncover a need for future investigation.

Second, given that we sampled women physicians both during and well after the menopausal transition, our survey results are likely subject to recall bias. Despite this, when controlling for current age in our regression analyses, the relationship between MRS score and our outcomes of interest remained statistically significant, suggesting that time since menopause did not significantly impact the relationship between symptom severity and the outcomes of interest. Furthermore, the MRS tool is validated for self-reported contemporaneous menopause symptom severity, as opposed to retrospective symptom reporting at their worst, as in our study. Although recall bias may influence our results, respondents’ quantitative and qualitative recollection of their menopausal symptom burden and their perceived impact on work can be of value. Qualitative responses, as highlighted in Box 1, also provide further confirmation that a more rigorous study is required to ensure that all women physicians feel supported in the workplace.

Lastly, the number of participants who took time off due to menopause symptoms was very small, and our data were therefore underpowered to explore the relationship between MRS score and absences from medicine.

Although these limitations must be considered when interpreting these data, the goal of this needs assessment was to shed light on how menopausal symptoms can potentially be impacting women physicians in Canada and identify future directions for research and the potential for interventions to improve working conditions for women physicians during the menopausal transition.

### 4.2. Future Directions

Women physicians are key contributors to the medical workforce in Canada. In 2019, there were over 15,000 women physicians in Canada under the age of 45, accounting for nearly 20% of the Canadian physician workforce [14]. As this age demographic approaches menopause, understanding the range of experiences and establishing supports and accommodations for those facing work-related challenges associated with the menopausal transition should be an urgent priority. Although our study emphasizes the widespread need for more support across all specialties and practice settings, the implementation of change must come at the institutional level. The MFC “Menopause in the Workplace” report includes concrete suggestions to improve culture and communication, policies, accommodations, and employee benefits that can guide medical organizations seeking to create menopause inclusive workspaces [13].

## 5. Conclusions

There is overwhelming evidence among physicians and non-physicians that the physical characteristics and psychosocial culture of the workplace make coping with menopausal symptoms harder. Although this exploratory, cross-sectional study may not represent the full spectrum of experiences of the menopausal transition among women physicians, our study does suggest that some Canadian women physicians may be coping with severe menopausal symptoms, often without support. Our study respondents reported working harder to persevere and maintain quality of care. It is prudent to reframe this important issue as not only an opportunity to support individual physicians, but also to improve productivity, job satisfaction, and gender and health equity in medicine. Future research on this topic, championed by workplaces and/or employers, may provide more representative data, elucidate the relationships between menopausal symptoms, productivity, and job satisfaction, and identify actionable changes relevant to specific work contexts. Our findings serve as a call-to-action for medical institutions and employers to explore and understand existing challenges faced by women physicians during the menopausal transition to promote and maintain the health of the women physician workforce.

## Figures and Tables

**Table 1:** Demographic characteristics of study participants, N = 217.

Demographic Characteristics	Frequency (%)
Current age	
≤40	3 (1.4%)
41–45	18 (8.3%)
46–54	101 (46.5%)
55–64	77 (35.5%)
≥65	18 (8.3%)
Believe to be	
Postmenopausal	156 (71.9%)
Perimenopausal	61 (28.1%)
Age of last menstrual period	
Pre-menopausal	61 (28.1%)
≤40	13 (6.0%)
41–45	14 (6.5%)
46–54	103 (47.5%)
55–64	26 (12.0%)
Location in Canada *	
Western	93 (42.9%)
Central	4 (1.8%)
Ontario region	89 (41.0%)
Quebec region	12 (5.5%)
Atlantic region	19 (8.8%)

* Location in Canada was categorized based on the Society of Obstetricians and Gynecologists of Canada (SOGC regions) as follows: Western region: British Columbia, Alberta; Central region: Saskatchewan, Manitoba, Northwest Territories, Yukon Territory; Ontario region: Ontario, Nunavut; Quebec region: Quebec; Atlantic region: Newfoundland and Labrador, New Brunswick, Nova Scotia, Prince Edward Island.

**Table 2:** Practice characteristics, N = 217.

Practice Characteristics	Frequency (%)
Specialty Type	
Surgical	65 (30.0%)
Non-surgical	149 (68.7%)
Missing	3 (1.4%)
Years in practice	
0–4	2 (0.9%)
5–9	7 (3.2%)
10–20	68 (31.3%)
>20	140 (64.5%)
Current work hours	
Part-time	67 (30.9%)
Full-time	150 (69.1%)
Type of practice	
Community	98 (45.2%)
Academic	56 (25.8%)
Both community and academic	63 (29.0%)
Practice geographic setting	
Urban or peri-urban	196 (90.3%)
Rural	18 (8.3%)
Remote	3 (1.4%)
Type of practice facility	
Hospital	61 (28.1%)
Clinic	70 (32.3%)
Both hospital and clinic	86 (39.6)
Job structure	
Group practice member	155 (71.4%)
Work independently	62 (28.6%)
Flexible Work Hours	136 (62.7%)
Night Shifts	127 (58.5%)

**Table 3:** Linear regression models for perceived negative work performance.

Simple Linear Regression			
**Predictors**	**B (SE)**	**95% CI**	***p*-value**
Constant	1.789 (0.160)	1.483–2.113	<0.001
Composite MRS score	0.086 (0.009)	0.068–0.104	<0.001
*Model summary: F (1,204) = 86.90, p < 0.001, R* *^2^ = 29.9%*			
**Multiple linear regression**			
**Predictors**	**B (SE)**	**95% CI**	***p*-value**
Constant	1.244 (0.340)	0.573–1.916	<0.001
Composite MRS score	0.082 (0.009)	0.064–0.101	<0.001
Age			
≤40	−0.042 (0.582	−1.190–1.106	0.943
41–45	0.349 (0.337)	−0.315–1.013	0.301
46–54	0.581 (0.260)	0.069–1.093	0.026
55–64	0.157 (0.263)	−0.362–0.675	0.552
≥65	Reference		
Practice type			
Community	0.304 (0.168)	−0.026–0.635	0.071
Academic	0.344 (0.197)	−0.046–0.733	0.083
Both community and academic	Reference		
Practice facility			
Hospital	−0.162 (0.179)	−0.515–0.190	0.366
Clinic	−0.066 (0.191)	−0.443–0.311	0.730
Both hospital and clinic	Reference		
Specialty			
Non-surgical	0.037 (0.156)	−0.271–0.345	0.814
Surgical	Reference		
Job includes overnight call/shifts	0.061 (0.150)	−0.235–0.358	0.683
Have flexible working hours	0.036 (0.147)	−0.253–0.326	0.804
*Model summary: F (12,190) = 9.05, p < 0.001, R* *^2^ = 36.4%*			

Note: B = regression coefficient, SE = standard error, CI = confidence interval.

**Table 4:** Linear regression models for perceived negative impact on promotional opportunities.

Simple Linear Regression			
**Predictors**	**B (SE)**	**95% CI**	***p*-value**
Constant	1.023 (0.159)	0.709–1.336	<0.001
Composite MRS score	0.083 (0.009)	0.065–0.101	**<0.001**
*Model summary: F (1,190) = 81.11, p < 0.001, R* *^2^ = 29.9%*			
**Multiple linear regression**			
**Predictors**	**B (SE)**	**95% CI**	***p*-value**
Constant	0.701 (0.340)	0.030–1.373	<0.001
Composite MRS score	0.083 (0.010)	0.065–0.102	<0.001
Age			
≤40	0.012 (0.574)	−1.120–1.144	0.983
41–45	0.425 (0.332)	−0.230–1.080	0.202
46–54	0.122 (0.258)	−0.387–0.632	0.637
55–64	−0.050 (0.261)	−0.565–0.466	0.849
≥65	Reference		
Practice type			
Community	0.208 (0.169)	−0.125–0.540	0.219
Academic	−0.128 (0.198)	−0.519–0.263	0.521
Both community and academic	Reference		
Practice facility			
Hospital	0.140 (0.182)	−0.220–0.500	0.444
Clinic	−0.236 (0.193)	−0.616–0.145	0.223
Both hospital and clinic	Reference		
Specialty			
Non-surgical	0.004 (0.157)	−0.305–0.314	0.977
Surgical	Reference		
Job includes overnight call/shifts	0.002 (0.152)	−0.299–0.302	0.991
Have flexible working hours	0.322 (0.149)	0.027–0.617	0.032
*Model summary: F (12,176) = 8.14, p < 0.001, R* *^2^ = 35.7%*			

Note: B = regression coefficient, SE = standard error, CI = confidence interval.

**Table 5:** Linear regression models for Job satisfaction.

Simple Linear Regression			
**Predictors**	**B (SE)**	**95% CI**	***p*-value**
Constant	5.367 (0.198)	4.978–5.757	<0.001
Composite MRS score	−0.032 (0.011)	−0.054–−0.009	**0.006**
*Model summary: F (1,214) = 7.84, p = 0.006, R* *^2^ = 3.5%*			
**Multiple linear regression**			
**Predictors**	**B (SE)**	**95% CI**	***p*-value**
Constant	6.025 (0.415)	5.206 to 6.844	<0.001
Composite MRS score	−0.024 (0.012)	−0.047 to −0.001	0.039
Age			
≤40	−1.068 (0.723)	−2.494–0.358	0.141
41–45	−0.726 (0.386)	−1.484–0.035	0.061
46–54	−0.914 (0.300)	−1.505–−0.322	**0.003**
55–64	−0.609 (0.303)	−1.208–−0.011	**0.046**
≥65	Reference		
Practice type			
Community	−0.056 (0.209)	−0.469–0.357	0.789
Academic	0.109 (0.242)	−0.369–0.587	0.655
Both community and academic	Reference		
Practice facility			
Hospital	0.344 (0.219)	−0.088–0.776	0.118
Clinic	−0.153 (0.237)	−0.620–0.314	0.519
Both hospital and clinic	Reference		
Specialty			
Non-surgical	−0.209 (0.192)	-0.588–0.171	0.280
Surgical	Reference		
Job includes overnight call/shifts	−0.161 (0.186)	−0.528–0.206	0.388
Have flexible working hours	0.205 (0.181)	−0.152–0.562	0.260
*Model summary: F (12,200) = 2.54, p = 0.004, R* *^2^ = 13.2%*			

Note: B = regression coefficient, SE = standard error, CI = confidence interval.

## Data Availability

The data presented in this study are available on request from the corresponding author due to privacy or ethical restrictions.
